# The new ambient-pressure X-ray photoelectron spectroscopy instrument at MAX-lab

**DOI:** 10.1107/S0909049512032700

**Published:** 2012-08-07

**Authors:** Joachim Schnadt, Jan Knudsen, Jesper N. Andersen, Hans Siegbahn, Annette Pietzsch, Franz Hennies, Niclas Johansson, Nils Mårtensson, Gunnar Öhrwall, Stephan Bahr, Sven Mähl, Oliver Schaff

**Affiliations:** a Division of Synchrotron Radiation Research, Department of Physics, Lund University, Box 118, 221 00 Lund, Sweden; b MAX IV Laboratory, Lund University, Box 118, 221 00 Lund, Sweden; c Department of Physics and Astronomy, Uppsala University, Box 516, 751 20 Uppsala, Sweden; d SPECS Surface Nano Analysis GmbH, Voltastrasse 5, 13355 Berlin, Germany

**Keywords:** ambient-pressure X-ray photoelectron spectroscopy, high-pressure X-ray photoelectron spectroscopy, X-ray photoelectron spectroscopy, *in situ*, operando, catalysis

## Abstract

The new instrument for near-ambient-pressure X-ray photoelectron spectroscopy which has been installed at the MAX II ring of the Swedish synchrotron radiation facility MAX IV Laboratory in Lund is presented. The new instrument, which is based on a SPECS PHOIBOS 150 NAP analyser, is the first to feature the use of retractable and exchangeable high-pressure cells. This implies that clean vacuum conditions are retained in the instrument’s analysis chamber and that it is possible to swiftly change between near-ambient and ultrahigh-vacuum conditions. In this way the instrument implements a direct link between ultrahigh-vacuum and *in situ* studies, and the entire pressure range from ultrahigh-vacuum to near-ambient conditions is available to the user. Measurements at pressures up to 10^−5^ mbar are carried out in the ultrahigh-vacuum analysis chamber, while measurements at higher pressures are performed in the high-pressure cell. The installation of a mass spectrometer on the exhaust line of the reaction cell offers the users the additional dimension of simultaneous reaction data monitoring. Moreover, the chosen design approach allows the use of dedicated cells for different sample environments, rendering the Swedish ambient-pressure X-ray photoelectron spectroscopy instrument a highly versatile and flexible tool.

## Abstract 2

The new instrument for ambient-pressure X-ray photoelectron spectroscopy at the Swedish synchrotron radiation facility MAX IV Laboratory is presented. The instrument is based on the use of a retractable and exchangeable high-pressure cell, which implies that ultrahigh-vacuum conditions are retained in the analysis chamber and that dual ambient pressure and ultrahigh-vacuum use is possible.

## 1. Introduction

Today, X-ray photoelectron spectroscopy (XPS) is one of the primary workhorses for electronic structure investigations of surfaces, vapours and liquids. The vast majority of all XPS instruments, whether home laboratory or synchrotron-based, operate under vacuum conditions, *i.e.* typically the sample environment pressure does not exceed 10^−6^ mbar. The primary rationale for operation in a vacuum is the strong inelastic electron scattering cross section in the kinetic energy range between a few and some hundreds of eV (see, for example, Schram *et al.*, 1966 ▶; Schutten *et al.*, 1966 ▶), which puts severe restrictions on the admissible path length of the photoelectrons through gas. However, the vacuum environment heavily limits the choice of sample; therefore, early on instruments were developed which could handle vapours by the introduction of differentially pumped gas cells into the vacuum chamber (Siegbahn *et al.*, 1969 ▶). The first dedicated XPS instruments for liquids (Siegbahn & Siegbahn, 1973 ▶) and surfaces in a vapour atmosphere (Joyner *et al.*, 1979 ▶) followed soon after. Since then, and until the beginning of the new century, the field of high-pressure X-ray photoelectron spectroscopy (HPXPS) [‘high’ implies pressures ‘only’ up to the mbar range, often instead called ambient-pressure X-ray photoelectron spectroscopy (APXPS)] developed continuously (Siegbahn, 1985 ▶; Littrell & Tatarchuk, 1986 ▶; Ruppender *et al.*, 1990 ▶; Grunze *et al.*, 1992 ▶), but slowly. It was with the advent of third-generation synchrotron radiation sources that APXPS experienced its breakthrough. These sources deliver a much higher photon flux than conventional X-ray and even second-generation synchrotron radiation sources. The high photon flux also implies a very large flux of photoelectrons, and APXPS measurements become much more feasible in spite of the large loss of photoelectron intensity owing to inelastic scattering in the ambient atmosphere. Moreover, pioneering groups at the ALS and BESSY were the first to use differentially pumped electrostatic lens systems in their electron energy analysers, which significantly increases the transmission of the high-pressure-adapted analysers (Ogletree *et al.*, 2002 ▶, 2009 ▶; Bluhm *et al.*, 2004 ▶). The two advances of new light sources and improved electron energy analysers have led to a strong increase in the number of publications using APXPS during the last ten years (for reviews see Salmeron & Schlögl, 2008 ▶; Knop-Gericke *et al.*, 2009 ▶). A couple of instruments available to external users are now in use at the ALS, BESSY and the NSLS and the trend goes towards the installation of further instruments at synchrotron radiation facilities around the world, a number of which will be opened to external users in the future. In addition there exist both further home laboratory-based (*e.g.* by Pantförder *et al.*, 2005 ▶) and private synchrotron-based instruments.

Here we present the new instrument for APXPS at beamline I511 on the MAX II ring, which was delivered in September 2010 to replace the previous ultrahigh-vacuum (UHV) end station for XPS and resonant inelastic X-ray scattering. The beamline (Denecke *et al.*, 1999 ▶) is an undulator-based soft X-ray beamline delivering photons in the energy range between 50 and 1500 eV. The light is monochromated by a plane-grating monochromator, and the light is refocused by a set of Kirkpatrick–Baez (KB) optics (Kirk­patrick & Baez, 1948 ▶). The APXPS instrument is connected to the beamline *via* a differential pumping stage similar to that employed at beamline I3 of the MAX III ring (*cf.* Urpelainen *et al.*, 2010 ▶). The efficient differential pumping stage allows measurements in gas pressures up to the 10^−5^ mbar range in the analysis chamber without any further instrumental measures.

## 2. The APXPS end station

**Figure 1:**
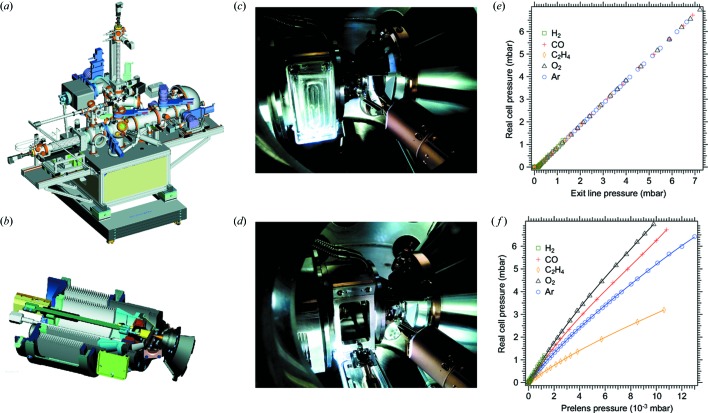
(*a*) Drawing of the HPXPS instrument at the MAX IV Laboratory. (*b*) Cross-section drawing of the high-pressure cell. At the front in green is the door which is opened for sample transfer. The nozzle is depicted in a golden colour and points at the sample (in brown). Sample heating is achieved through the wall behind the sample by electron bombardment. The double set-up of bellows makes it possible to move the sample during measurement. (*c*) High-pressure cell during approach to the analyser. (*d*) Sample loading into the high-pressure cell docked to the analyser. (*e*) Relationship between real absolute and measured pressure in the gas cell for a number of gases. (*f*) Relationship between real absolute pressure and pressure in the pre-lens of the PHOIBOS 150 NAP analyser.

In contrast to other APXPS set-ups our instrument (Fig. 1*a* ▶) is capable of dual and combined ambient pressure and UHV use. This is made possible by the use of a retractable ‘high-pressure cell’ [Figs. 1(*b*)–1(*d*) ▶], which can be docked to the front aperture of the SPECS PHOIBOS 150 NAP analyser (see http://www.specs.de/) for ambient pressure use. Only the cell is filled with gases or vapours, while the analysis chamber retains a pressure of around 10^−6^ mbar even when the pressure is increased to 10 mbar in the cell. After retraction of the high-pressure cell into a separate chamber, UHV conditions are achieved typically within less than an hour. For ambient-pressure operation the transferable sample is loaded into the high-pressure cell, which before gas exposure is locked off from the surrounding UHV of the analysis chamber by means of a door operated by the transfer wobblestick. The high-pressure cell carries an exchangeable nozzle which upon docking constitutes the interface towards the pre-lens stage of the analyser. As in other APXPS instruments the nozzle and further apertures in the electrostatic lens system of the analyser allow differential pumping, which brings the pressure down to 5 × 10^−8^ mbar or better at the multichannel plate detector and thus allows ambient-pressure use (Ogletree *et al.*, 2002 ▶). Presently available nozzle orifices have diameters of 0.3 mm and 1 mm. For reference, with the 1 mm nozzle, at 2 mbar O_2_ pressure in the high-pressure cell, the pressure in the analysis chamber was measured to be 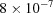
 mbar, pre-lens 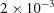
 mbar, first pumping stage 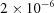
 mbar, second pumping stage 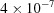
 mbar, and detector 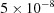
 mbar.

A gas system with gas cleaning facilities supplies mixtures of presently up to three gases to the cell. The flow is regulated by separate flow controllers for each of the gases. The gas exit line of the cell, connected to a roughing pump, contains a pressure controller, and thus pressure, gas flow and gas mixture can be controlled independently. Both the gas supply and gas exit lines are connected to a quadrupole mass spectrometer (QMS) *via* leak valves, so that the gas composition before and after sample contact can be analysed. The sample temperature is measured with a chromel-alumel thermocouple wire pair mounted on the transferable sample holder. The thermocouple junction is in direct contact with the sample, while, with the sample in place for measurement, the other ends of the wires connect to cables to outside feedthroughs *via* a contact on the sample holder. Thus, the sample temperature is known to within the accuracy of the thermocouple measurement. Heating is achieved through electron bombardment of the vacuum side of the wall behind the sample seat; a water-cooling loop ensures that only the seat, and not the surrounding material, is heated.

The pressure in the cell is measured by a Pirani gauge on the exit line. As is seen from Fig. 1(*e*) ▶, this pressure scales linearly with the real pressure in the cell as evaluated by an absolute-pressure transducer. For pressure calibration one can also use the pressure measured in the pre-lens (Fig. 1*f* ▶). This pressure, however, depends on the type of gas used as an effect of different orifice pumping speeds for different gases. The advantage of using the pre-lens pressure is that it can be used for real cell pressures down to 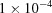
 mbar, while the Pirani is limited to minimum pressures of around 0.1 mbar. This implies that the instrument as a whole is capable of *in situ* measurements over the entire pressure range from UHV to mbar, with measurements up to 10^−5^ mbar being performed directly in the analysis chamber and higher pressures in the high-pressure cell.

A particular feature of the chosen design concept with high-pressure cells is also that the cells can be exchanged easily. This allows for custom-made cells for different applications and chemical reactions. For example, we are in the course of developing a cell for APXPS measurements on liquids, which should go into operation later this year (Siegbahn *et al.*, 2012 ▶). It is also conceivable to develop cells for, for example, corrosive gases, high-temperature applications, *in situ* electrochemical measurements, *etc*. Thus, the design concept opens for a highly flexible use of the APXPS instrument.

## 3. Proof-of-principle measurement

**Figure 2:**
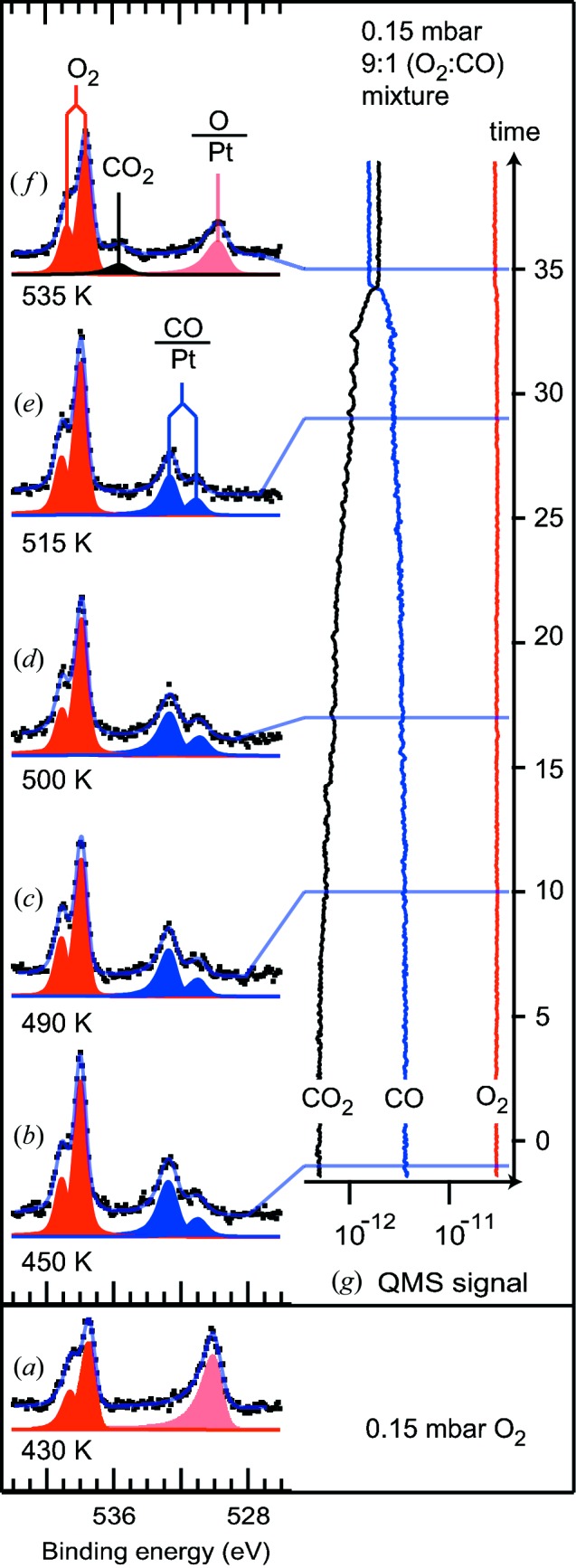
(*a*) O 1*s* X-ray photoelectron spectrum of Pt(111) in 0.15 mbar O_2_ at 430 K. (*b*)–(*f*) O 1*s* X-ray photoelectron spectra of Pt(111) in a 0.15 mbar 9:1 O_2_:CO mixture when the crystal is heated from 450 to 535 K. (*g*) QMS signal from CO, O_2_ and CO_2_ acquired simultaneously with the XPS data.

We have tested the set-up in a CO oxidation experiment over Pt(111). The data are reproduced in Fig. 2 ▶. Initially the Pt(111) single crystal was exposed to 0.15 mbar pure di-oxygen at 430 K [panel (*a*)]. Under these conditions the O 1*s* spectrum is dominated by a component at 530.0 eV binding energy assigned to atomic oxygen adsorbed on the surface (Björneholm *et al.*, 1994 ▶) and two components at 537.4 eV and 538.5 eV both assigned to molecular oxygen in the gas phase. Subsequently, CO was mixed into the gas at a 9:1 O_2_:CO ratio and the same total pressure of 0.15 mbar. Panels (*b*)–(*f*) of Fig. 2 ▶ show the O 1*s* region for this gas mixing ratio and different sample temperatures between 450 and 535 K. The atomic oxygen component at 530.0 eV disappears as soon as CO is mixed into the gas, and instead two new components are observed at 531.0 eV and 532.6 eV, consistent with CO adsorbed in bridge and on-top sites, respectively (Björneholm *et al.*, 1994 ▶). These O 1*s* fingerprints of CO persist until a sample temperature of 515 K is reached. In the O 1*s* spectrum measured at 535 K the two CO adsorbate-induced components are absent, and instead the atomic oxygen component at 530.0 eV reappears together with a new component at 535.6 eV, which is due to CO_2_ in the gas phase.

The evolution of the chemical reaction was also followed using the mass spectrometer mounted at the outlet of the high-pressure cell (Fig. 2*g* ▶). Clearly, CO_2_ production is low at temperatures below 515 K; adsorbed CO completely covers the surface and limits the surface’s reactivity. Still, there is a steady increase of the CO_2_ signal and a corresponding decrease in CO signal. This low-temperature activity is in line with the previously reported onset temperature for CO oxidation over Pt(111) of 305 K (Burnett *et al.*, 2004 ▶). Moreover, low-temperature reactivity over surface steps is further enhanced at the oxygen pressures used here (Lewis *et al.*, 2005 ▶), which might further contribute to the measured catalytic activity below 515 K.

The large change of the CO_2_ and CO, and indeed also O_2_, mass spectrometer signals at 535 K shows that the production of CO_2_ is increased dramatically at this sample temperature. This is the same temperature at which the CO-related peak in the O 1*s* spectrum disappears and is replaced by a peak due to chemisorbed oxygen. Thus, although the reaction proceeds in a Langmuir–Hinshelwood mechanism (Ertl, 1994 ▶), no adsorbed CO is visible anymore in the O 1*s* spectrum (Fig. 2*f* ▶). The reactivity is so high that the average CO coverage is too low to be observed; only the atomic oxygen coverage is seen for this highly active phase.

## 4. Summary and conclusions

This example shows that the new set-up can be used for performing APXPS experiments and, moreover, that it is possible to correlate the XPS results to mass spectra data provided by the mass spectrometer which is connected to the exit of the high-pressure cell. Hence, the instrument does not only allow XPS measurements on the same sample under UHV and near-ambient-pressure conditions, it also offers the user the additional dimension of simultaneous reaction data monitoring.

At present, the focus of the beamline is not optimised for our set-up. Indeed, the light spot is around 2–3 mm in diameter, which compares unfavourably with the focus size of the analyser, which for the 0.3 mm aperture is around 0.2 mm × 0.2 mm. A new beamline, the SPECIES beamline, has received funding, which features an elliptically polarising undulator, a collimated plane-grating monochromator, and optimised refocusing optics with a spot size of 0.1 mm. The SPECIES beamline will further improve the opportunities for APXPS experiments at the MAX IV Laboratory in the near future.
